# From diversity to function: microbiome-mediated plant growth promotion, secondary metabolism, and antimicrobial resistance in *Rauwolfia serpentina*

**DOI:** 10.3389/fbinf.2026.1796770

**Published:** 2026-07-14

**Authors:** Vrishali Rajendra Bankar, Shilpa Samir Chapadgaonkar, Kausik Bhattacharyya, Prabha K

**Affiliations:** 1 Department of Biosciences and Technology, Dr. Vishwanath Karad, MIT World Peace University, Pune, Maharashtra, India; 2 ICAR-Directorate of Floriculture Research, Pune, Maharashtra, India

**Keywords:** metagenomics, microbiome, biosynthetic gene clusters, plant growth promoting rhizobacteria (PGPRs), plant-microbe interactions, *Rauvolfia serpentina*

## Abstract

**Introduction:**

This study presents the first metagenomic analysis of the root and rhizosphere microbiomes of *Rauvolfia serpentina,* an endangered medicinal plant. Metagenomic sequencing and bioinformatics analysis were used to characterize the diverse microbial communities and their functional attributes to assess the ecological and biotechnological potential of this plant-associated microbiome.

**Methods:**

High-throughput Illumina sequencing and bioinformatics analysis were used to profile the microbial communities. Functional annotation was performed to identify plant growth-promoting traits using PLABASE, to predict pathways for the biosynthesis of novel bioactive compounds using antiSMASH, and to identify antimicrobial resistance genes using ResFinder.

**Results:**

The analysis revealed highly diverse microbial communities in both habitats, predominantly composed of Pseudomonadota, Bacillota, and Actinomycetota, with minor but consistent contributions from archaea and eukaryotes. Functional annotation identified extensive PGPTs, including genes associated with phosphate solubilization, nitrogen fixation, siderophore-mediated iron acquisition, and stress tolerance. The rhizosphere microbiome exhibited greater metabolic versatility and stress tolerance, characterized by a higher copy number of heavy metal efflux pumps, whereas the root microbiome was enriched in genes involved in plant hormone regulation and plant-microbe interactions. A diverse array of non-ribosomal peptide synthase, polyketide synthase, and lasso peptide pathways were predicted, underscoring the potential to produce novel bioactive compounds. These distinct functional profiles demonstrates that the protected root endomicrobiome specializes in plant signalling and nutrient assimilation, while the rhizosphere microbiome, facing higher competition, specializes in nutrient acquisition and stress resilience.

**Conclusion:**

These findings provide novel insights into the ecological specialization and biotechnological potential of the *R. serpentina* microbiome, offering significant implications for the sustainable utilization and conservation of this endangered medicinal plant.

## 1 Introduction

*Rauvolfia serpentina* (Indian snakeroot) is a medicinal plant known for its indole alkaloids has traditionally been used for the treatment of several types of ailments such as hypertension and psychiatric disorders (Malviya and Sason, 2016; Shah et al., 2020; El-Saadony et al., 2025). However, overexploitation, habitat loss, and unsustainable harvesting techniques have rendered *Rauwolfia serpentina* endangered, as listed by the International Union for Conservation of Nature (IUCN) (Gowthami et al., 2021). This calls for urgent efforts for the conservation of this medicinally significant plant.

Recent advances in plant microbiome research have demonstrated that rhizospheric and root-associated microbial communities play critical roles in plant growth and health (Lazcano et al., 2021; Pantigoso et al., 2022). These microbiomes facilitate nutrient acquisition, enhance stress tolerance, and contribute to disease suppression, thereby supporting plant survival in challenging environments (Khan, 2023). This niche of the plant microbiome is distinct and can be explored as a valuable resource for isolating microorganisms with distinct metabolic capabilities. Moreover, plant microbiome can be used effectively as a benchmark for designing bio-inoculants for improving plant productivity and health (Ren et al., 2025). Understanding the taxonomic and functional diversity of microbial communities associated with *R. serpentina* can provide essential information for the conservation of this endangered plant. Previous studies on other plants have demonstrated that bacteria, particularly Pseudomonadota, Bacillota, and Actinomycetota, dominate both the rhizosphere and root endosphere. These bacteria exhibit several plant growth-promoting traits, such as nitrogen fixation, phosphate solubilization, siderophore production, and phytohormone modulation, and are potential sources of novel bioactive compounds for agriculture and medicine (Cheng et al., 2019).

Previous studies on *R*. *serpentina* have largely focused on its pharmacology, phytochemistry, and multi-omics to elucidate pathways of alkaloid biosynthesis and its regulation (Prakash et al., 2016; Tyagi et al., 2026). However, our understanding of its associated microbial communities remains fragmented. Parallelly, culture-dependent studies have successfully isolated endophytes and studied their growth-promoting potential (Pal and Paul, 2020; Lata and Gond, 2025; Shrivastava et al., 2025). It needs to be noted that, these approaches fail to understand the largely unculturable microbiome of these specialized niches. This study presents the first comprehensive metagenomic analysis of the *R. serpentina* root and rhizosphere microbiomes, offering an extensive view of their taxonomic diversity, functional gene repertoire, and biosynthetic capacity. By elucidating these microbial signatures, our findings provide a critical foundation for developing bio-inoculant based conservation processes, and bioprospecting for novel natural products.

## 2 Materials and methods

### 2.1 Sample collection and processing

Rhizospheric soil (within 1–7 cm from plant roots and 15–20 cm depth underground) and root samples were collected from Ahmednagar, Maharashtra 41,400, India, in August 2024, following standard plant species identification procedures. Authentication certificate of the plant species identification was carried out from Agarkar Research Institute Pune (Report AUTH 24–131) (shared as Supplementary Material). The samples were stored at −80 °C until further analysis (Liu et al., 2024).

Triplicate samples from *R. serpentina* root and its rhizosphere were processed, and equal amount of DNA from each sample was pooled into a single library for metagenomic sequencing. Pooling was performed to maximize sequencing depth and obtain a representative community profile from each habitat, a strategy used in prior exploratory metagenomic surveys (Adebayo et al., 2025; Dai et al., 2025). While this approach sacrifices biological replicate-level variance estimation, it provides a broad taxonomic and functional landscape of each environment, which was the primary aim of this study.

### 2.2 Genomic DNA extraction and sequencing

**FIGURE 1:**
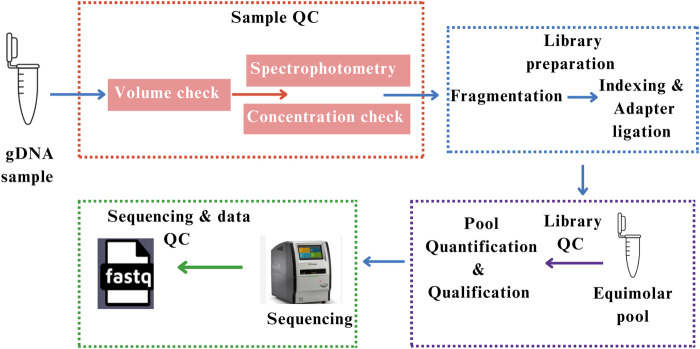
Sequencing workflow.

Root samples for DNA extraction were surface sterilized using 1% v/v sodium hypochlorite for 2 min followed by 70% v/v ethanol for 1 min, followed by 2-3 washes with sterile distilled water (Szkuta et al., 2015; Khalil et al., 2021; Bankar and Chapadgaonkar, 2025; de Silva Wijeyeratne and Gweon, 2025). Subsequently, genomic DNA of root and rhizosphere soil was isolated using the QIAamp DNA mini kit (Qiagen). The quality of the isolated DNA was determined by agarose gel electrophoresis and spectrometry (A_260_/A_280_ ratio). 250 ng of DNA from each pooled sample was used for library preparation using the QIASeq FX DNA kit (Qiagen) to fragment and obtain adapter-ligated and indexed libraries. The indexed library was sequenced using 300-cycle paired-end Illumina sequencing (Illumina NextSeq 2000) (Figure 1), generating approximately 31.4 million raw reads per pooled root sample with 4.62 Gb of sequencing data and 22.8 million raw reads per pooled soil sample with 3.36 Gb of sequencing data.

### 2.3 Metagenomic analysis

Quality of raw FASTQ files was analysed using FastQc v0.11.9 (Wingett and Andrews, 2018). The fastp tool (v0.12.4) (Chen et al., 2018) was used to remove adapter contamination. Reads with a quality score of Q30 or higher (indicative of a base-call accuracy probability of 99.9%) were used for downstream processing and the data was submitted to NCBI database. It can be accessed through NCBI accession number SAMN50861700, SAMN50861701 (https://submit.ncbi.nlm.nih.gov/subs/sra/SUB15545522/overview). Metagenome assemblies generated using MEGAHIT (Li et al., 2015), served as input for CCMetagen for microbial classification and identification, and to identify acquired antibiotic resistance genes using ResFinder (Marcelino et al., 2020; Florensa et al., 2022). Genome binning and refinement were performed using Anvi’o (Eren et al., 2015). Bin annotation was performed using Prokka (Seemann, 2014). These annotated files then served as input for PLaBAse (for detecting PGPR traits), antiSMASH 8.0, and PRISM analysis (to identify genes responsible for secondary metabolite production (Skinnider et al., 2020; Patz et al., 2021; 2024; Blin et al., 2025).

Random Forest (RF) classification was carried out for each gene category using the scikit-learn implementation to find the distinguishing genes of nitrogen and phosphorus metabolism between root and soil-derived metagenome-assembled genomes (MAGs). Each of the 12 recovered bins were treated as an individual observation. Gene copy numbers for nitrogen and phosphorus metabolism genes were normalized to relative abundance per 1,000 total annotated genes within each bin to account for differences in genome size and sequencing depth. Genes with zero abundance across all 12 bins were excluded. Relative abundances were z-score standardized across bins for each gene and visualized as a hierarchically clustered heatmap. Bins were ordered by environment of origin while genes columns were hierarchically clustered to reveal co-abundance patterns.

**FIGURE 2:**
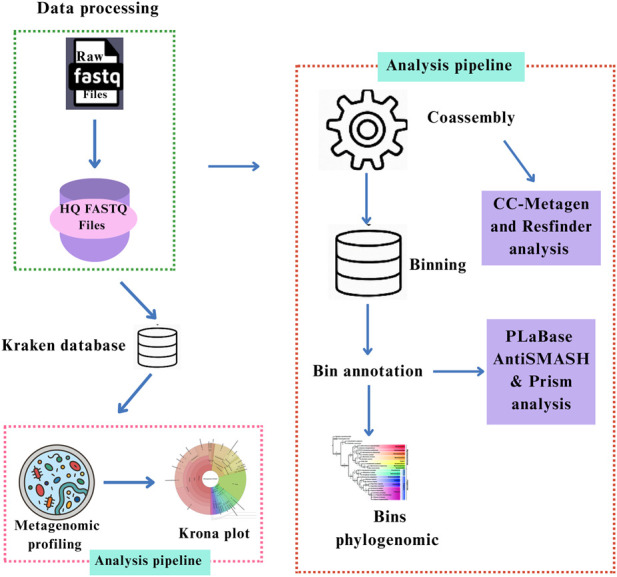
Analysis workflow.

Phylogenomic analysis was performed using GTO-tree (Lee, 2019). For metagenomic annotation and profiling, raw reads were analyzed using Mini-Kraken (Wood and Salzberg, 2014). Metagenomic profiling, representing taxonomic levels from super kingdom to family based on NCBI taxonomy, was visualized as Krona plots (Supplementary Figure S1) (Figure 2), (Table 1).

**TABLE 1:** Analytical tools used in this study.

Tool	Type of input file used for analysis	Application
PLaBAse	Bin annotation output files	Detection of PGPR traits
AntiSMASH 8.0 & prism	Bin annotation output files	Detection of biosynthetic gene clusters (secondary metabolites)
CC-metagen	Metagenome assembly output files	Identification of eukaryotes and prokaryotes in metagenomic data
ResFinder	Metagenome assembly output files	Identification of acquired antibiotic resistance genes

## 3 Results

Metagenomic assembly of the root sample yielded 108,612 contigs with total assembly size 280.52Mb, while the soil sample yielded 73,217 contigs with total assembly size of 54.17Mb, from which 8,244 contigs (12.89 Mb total) met the strict coverage thresholds for downstream profiling in anvi’o. To ensure high-fidelity downstream functional and taxonomic profiling within the anvi’o platform, a conservative minimum contig length threshold of 1000bp was strictly applied. This filter retained 108,612 contigs for the root sample, which were partitioned into 7 high-resolution metagenome assembled bins spanning 220.01Mb, with mean bin GC content ranging from 34.16% to 64.43% and a maximum contig N50 of 20,862 bp. For the soil sample assembly, the filter retained 8,244 contigs which clustered into 5 distinct bins showing genomic GC contents between 42.96% and 65.77% and contig N50 values ranging from 1,321 bp to 2,435 bp (Supplementary Table S1).

### 3.1 Microbial diversity and taxonomic distribution across rhizosphere-root compartments

Rhizospheric soil and root microbial communities predominantly composed of prokaryotic taxa. Bacteria constituted the major fraction, with Proteobacteria, Firmicutes, and Actinobacteria (Pseudomonadota, Bacillota, and Actinomycetota) representing about 96% of the total microbial community. Other bacterial groups, such as Cyanobacteria, Bacteroidetes, Gemmatimonadetes, Acidobacteria, and Planctomycetes, were also identified, highlighting the complex community architecture. Low abundance of archaeal sequences, primarily from Thaumarchaeota, Euryarchaeota, and Crenarchaeota, were detected mainly in rhizospheric soil but were not detected in root endomicrobiome. This indicates potential exclusion mechanisms for archaeal colonization in the root tissue. Viral sequences were also present, albeit at extremely low levels.

**FIGURE 3:**
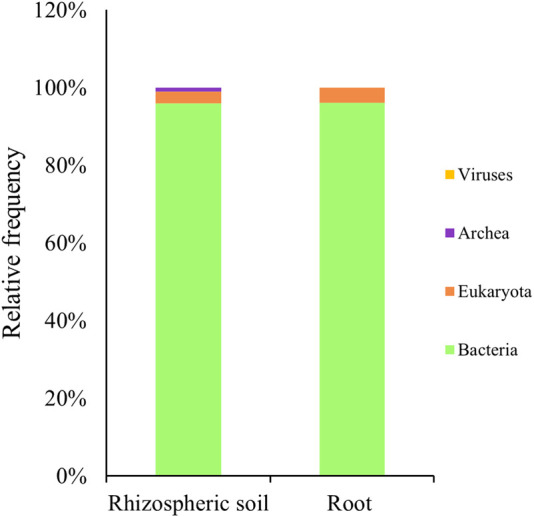
Relative taxonomic abundance at domain level.

**FIGURE 4:**
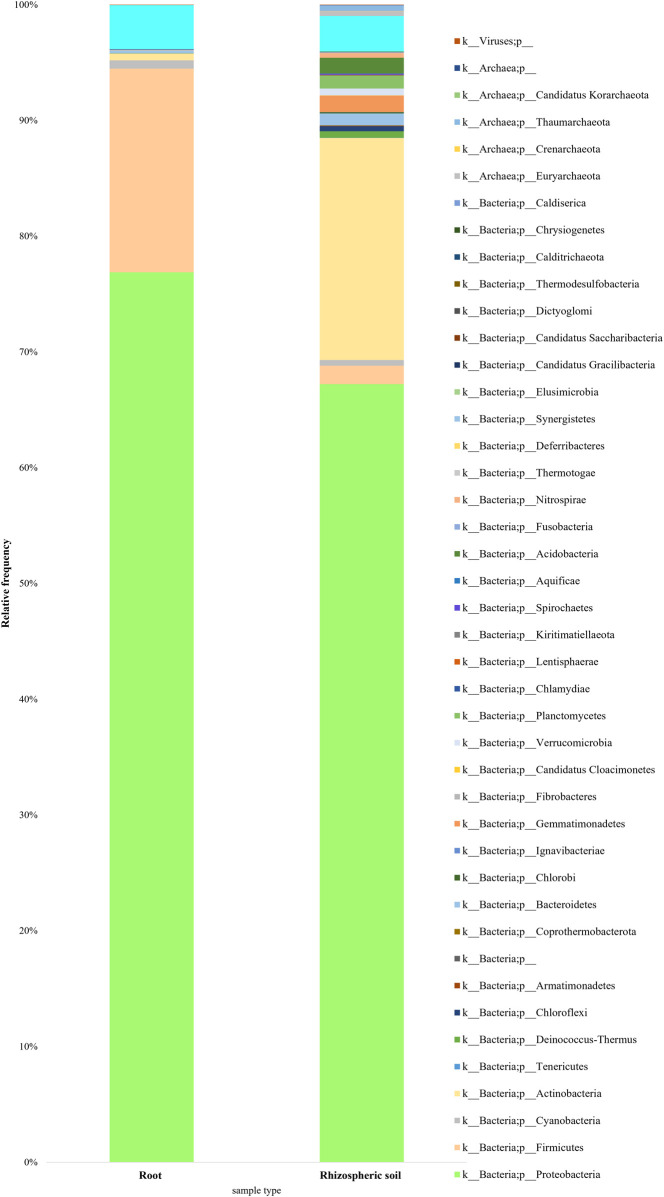
Relative taxonomic abundance at phylum level.

At the phylum level, both environments were dominated by Proteobacteria (the predominant green fraction in the taxonomic profiles, as shown in (Figures 3, 4), followed by substantially lower abundances of Firmicutes, Actinobacteria, and other bacterial phyla. The very low detectable viral sequence quantities in both compartments suggests either methodological limitations in viral detection or genuinely low viral abundance in this plant-soil system. The nearly identical community structures observed between these two microenvironments indicate that the rhizosphere serves as an effective reservoir, establishing the foundational microbial community composition before root colonization. The root environment appears to exert minimal additional selective pressure beyond the exclusion of archaea. Alpha and beta diversity results were computed however since samples were pooled into one they cannot compare statistically (Supplementary Figure S2).

### 3.2 Core microbiota and niche-specific taxa in root and rhizosphere

**FIGURE 5:**
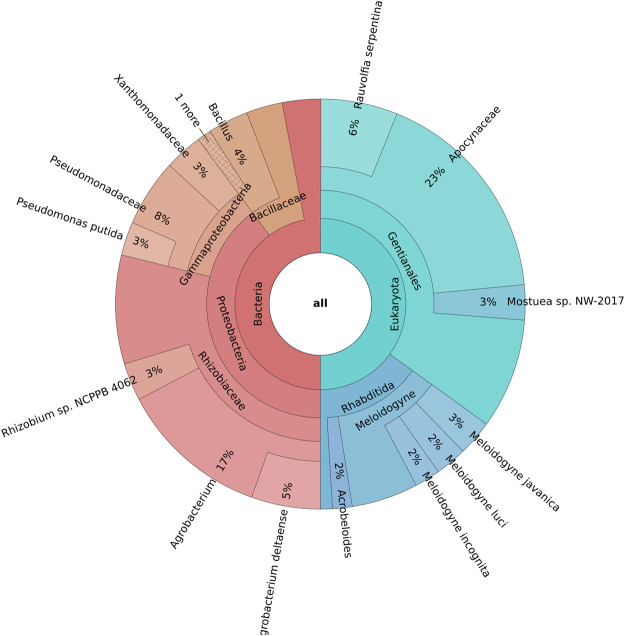
CC Metagen visualization of taxonomic composition of root microbiome.

**FIGURE 6:**
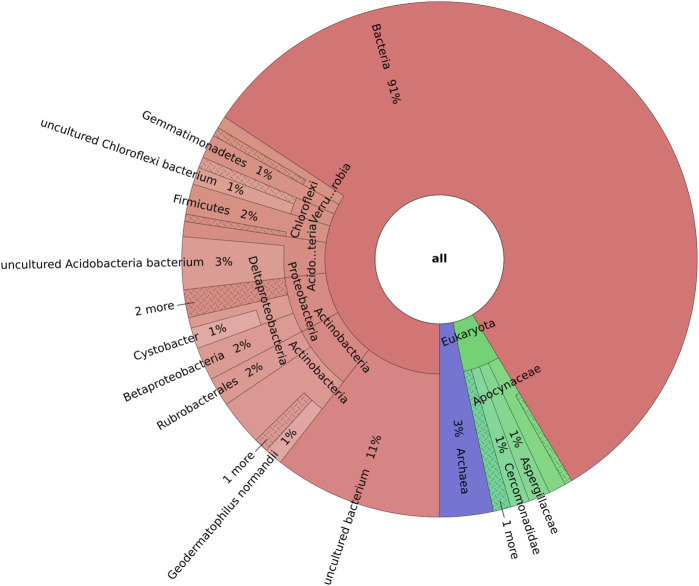
CC Metagen visualization of taxonomic composition of rhizospheric microbiome.

CCMetagen analysis revealed both overlapping and distinct communities in the root and the rhizosphere. Both environments were dominated by bacterial taxa, with Proteobacteria and Firmicutes prominently represented (Figures 5, 6). Key genera, such as *Agrobacterium*, *Pseudomonas*, and *Bacillus*, were detected in both niches, supported by high-confidence gene annotations for ribosomal RNA and functional enzymes. However, the soil sample exhibited greater overall bacterial diversity, including substantial representation from Actinobacteria and Acidobacteria, and a higher abundance of unclassified genera and species. This highlights the broader taxonomic richness typical of rhizospheric soil microbiomes. Whereas, the root sample was characterized by a more defined eukaryotic component, particularly plant-parasitic nematodes of the genus *Meloidogyne*. These were detected in both environments but with higher coverage and diversity in the roots, consistent with their ecological association with plant roots. Collectively, these findings demonstrate a shared core microbiota between root and rhizospheric soil. The soil harbours greater diversity, while the root niche consists of selected microbial species and plant-parasitic nematodes.

### 3.3 Bin annotation and phylogenomic analysis

Seven bins identified in the root sample accounted for 220,006,440 nucleotides, representing 78% of all nucleotides stored in the contigs database and 78.43% of nucleotides stored in the profile database. Similarly, five bins identified in the rhizospheric soil sample accounted for 12, 888, 870 nucleotides, representing 24% of all nucleotides stored in the contigs database and 100% of nucleotides stored in the profile database. The annotated files were subsequently used for functional analysis. Phylogenomic analysis of each bin from both samples was also performed using GTO-tree (Lee, 2019), with results provided as Supplementary Material. The low bin recovery from soil is consistent with the extraordinary taxonomic richness of soil environments, where even deeply sequenced metagenomes typically recover only a fraction of community members as high-quality metagenomically assembled genomes. The complexity and evenness of soil communities means that many organisms fall below the sequencing depth threshold required for reliable contig assembly and binning (Lemos et al., 2011; Johansen et al., 2025).

### 3.4 Functional genomic repertoires underpinning plant growth promotion

PLaBAse analysis (Patz et al., 2021; 2024) of the *R. serpentina* root microbiome revealed a genetically diverse and metabolically robust microbial community endowed with various plant growth-promoting traits (PGPTs), indicating symbiotic potential. Across all bins, genes involved in nutrient cycling, especially nitrogen metabolism, were consistently represented. Genes related to phosphorus acquisition and solubilization, as well as sulphur metabolism, illustrating the robust cycling of these essential nutrients. The microbial community demonstrated notable metabolic versatility in carbon utilization and energy generation and a broad array of glycoside hydrolases, which facilitates for the degradation of complex plant-derived carbohydrates. Iron acquisition is a prominent PGPT, with multiple bins harbouring diverse siderophore synthesis and transport systems. The community also displayed substantial stress resilience, with abundant genes for oxidative stress response, heavy metal resistance, and osmotic balance. These functional insights were derived from genome-recovered bins.

#### 3.4.1 Nitrogen cycling cooperation

**FIGURE 7:**
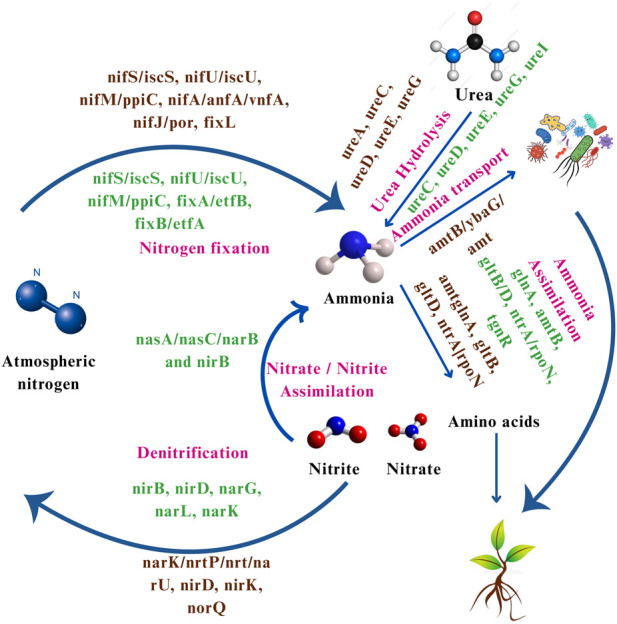
A comprehensive schematic of nitrogen cycling in the root and rhizospheric soil microbiome. The diagram illustrates key metabolic pathways including Nitrogen fixation, Denitrification, ammonia assimilation, Nitrate/Nitrite assimilation, Urea Hydrolysis, ammonia transport. (Note- Gene labels are color-coded to indicate their primary location: green for the root microbiome and brown for the soil microbiome. This figure demonstrates the robust and multifaceted nitrogen metabolism supported by the microbial communities at the plant interface).

**FIGURE 8:**
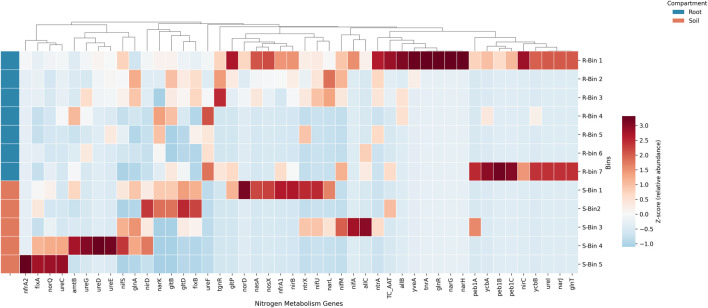
Hierarchically clustered heatmap of gene level relative abundance of nitrogen metabolism in root and rhizosphere microbiomes.

Hierarchical clustering of nitrogen metabolism genes (10) revealed distinct co-abundance gene clusters, with several genes (*ureF*, *fixA*, *nifS*) showing consistently elevated z-scores across root bins compared to soil bins, while denitrification-related genes (*nirD*, *norQ*) showed elevated values in a subset of soil bins (Figure 7). Nitrogen cycling was strongly represented in root microbiome, with key genes for ammonia transport, multiple nitrate/nitrite reduction and nitrogen fixation pathways. The root endomicrobiome demonstrated highly coordinated regulatory and assimilation systems, evidenced by presence of nitrogen sigma factor *ntr*A*| rpo*N and *tgn*R. The endomicrobiome emphasized assimilation and intracellular balance with genes for glutamine/glutamate metabolism (*gln*A, gltB/D) that encode glutamine synthase (GS)- Glutamate synthase (GOGAT) and transporters that funnel reduced nitrogen into amino acid pools. Interestingly, while *gln*A was distributed in both compartments, gltB and gltD showed greater presence in root endomicrobiome. The presence of urease subunits (*ure*C, *ure*F and *ure*G) further support the capacity for urea hydrolysis and additional nitrogen provisioning within the endosphere. The rhizospheric soil microbiome, was enriched with alternative components for nitrogen acquisition and redox activity interfacing the soil nitrogen pool. For instance, the nitrate/nitrite transporter *nar*K|nrtP|nrt|*nar*U showed consistent presence across the rhizosphere microbiome, aiding substrate uptake, whereas nitrite reduction (*nir*B and regulatory *nar*L) was predominantly enriched within root microbiome. Associative nitrogen fixation (*nif*S|*isc*S, *nif*U|*isc*U, and *nif*M|*ppi*C) was encoded against both the niches, indicating shared capacity for atmospheric nitrogen conversion, which is typically favoured by rich carbon root exudates and lower oxygen concentration at the root interface. The net effect can be summarized as rhizosphere microbiome acquire and transport nitrogenous substrates from the soil matrix, and the root endomicrobiome assimilates, regulates, and shuttles bioavailable nitrogen as per the host demand (Table 2) (Figures 7, 8).

**TABLE 2:** Nitrogen metabolism genes predicted by PLaBAse functional annotation pipeline.

Root microbiome	Rhizosphere microbiome
Nitrogen fixation: nifS/iscS, nifM/ppiC, nifA/anfA/vnfA	Nitrogen fixation: nifS/iscS, nifU/iscU, nifJ/por, fix, nifA/anfA/vnfA, nifM/ppiC, fixA/etfB and fixC
Nitrate/nitrite reduction and Transport: narG/narZ/nxrA, nasA/nasC/narB, nirB, nirD, narK, narL	Nitrate/nitrite reduction& Transport: nark/nrtP/nrt/narU, nirB, nirD, nreC, norQ, nirK
Nitrogen assimilation and Regulation: glnA, , gltB, gltD, gltP|gltT, fnr, ntrA|rpoN, tgnR, TC_AAT|yifK	Nitrogen assimilation and Regulation: glnA, gltB, gltD, ntrA|rpoN
Ammonia transport: amtB|ybaG| amt	Ammonia transport: amtB|ybaG| amt
Urease subunits: ureC, ureD, ureE, ureG, ureI	Urease subunits: ureA, ureC, ureD, ureE, ureG

#### 3.4.2 Phosphate solubilization and uptake

**FIGURE 9:**
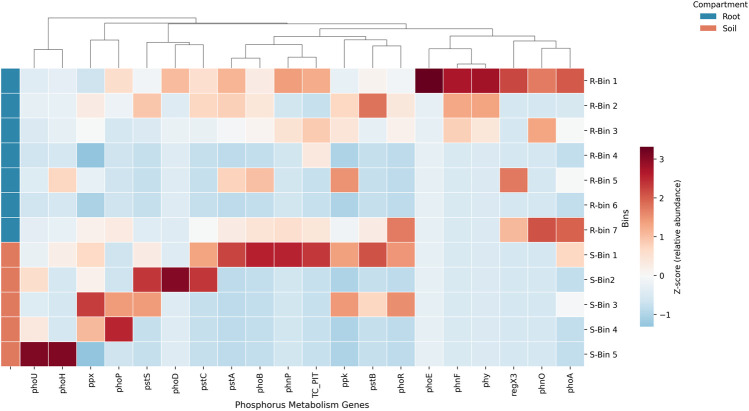
Hierarchically clustered heatmap of gene level relative abundance of phosphorous metabolism in root and rhizosphere microbiomes.

For phosphorus metabolism genes (Figure 9), ppx, *phoU*, and *pstS* clustered together and showed comparatively higher z-scores in several soil bins relative to root bins, while *phn* cluster genes were predominantly elevated in root bins. The *pho*A and *pho*D genes, which encode alkaline phosphatases, were identified in both rhizospheric as well as in the endophytic community. These phosphatases increase the bioavailability of organic phosphorus. Furthermore, the components of the pyrroloquinoline quinone biosynthesis (PQQ) operon, including *pqq*C, *pqq*D and *pqq*E (along with associated marker elements *arg*D|*pqq* and *pqq*L|*ydd*C) were well represented in both microbiomes. The biosynthetic product of this operon, PQQ, serves as an essential redox cofactor for alternative bacterial dehydrogenases that drive the production and secretion of organic acids (Such as gluconic acid), which lower the soil pH, resulting in solubilization of inorganic phosphorus. Phosphate utilization-*phn*F, *phn*O genes were found within the root microbiome, and were absent in the rhizospheric soil microbiome. The genes such as *ppx/ppx-gpp*A, which encode polyphosphohydrolases required for degradation of polyphosphate storage molecules were heavily enriched within the endophytic niches. This reveals a functional focus on mobilization and recycling of host derived phosphate storage compounds within the root tissues. Additionally, core components of the *pho regulon* which is a global regulatory network in bacteria managing phosphate starvation and stress adaptation was broadly identified across the microbiome, such as *pho*H, *pho*U, *pho*Y showing widespread redundancy across both microbiomes. Overall, the rhizospheric microbiome maintains robust genetic capabilities for extracellular phosphorus acquisition, mineral mobilization and structural stress adaption, whereas endophytic community relies mostly on conversing and transforming host derived phosphorous sources (Table 3).

**TABLE 3:** Phosphate Solubilization & Transport genes predicted by PLaBAse functional annotation pipeline.

Root microbiome	Rhizosphere microbiome
Phosphorus solubilization and uptake: phnO, phnP, phnF, phoA, phoD, phy|phyA|phyB|phyC, phoE, ppx|ppx_gppA, pstA, pstB|phoT, pstC|phoW, pstS|phoS, phoU|phoY, TC_PIT, phoB, phoP|phoB1, regX3, ppk, phoH, phoR pqqL|yddC, pqqE, pqqB|pqqG, pqqC, pqqD, argD|pqqI	Phosphorus solubilization and uptake: phoA, ppx/ppx_gppA phnE, phoD, pstS/phoS, TC_PIT, pstA, ppa, pstB/phoT, phoU/phoY, phoH, phoR, pstC|phoW, phoB, phnP, pstA, phnD, phoP|phoB1 argD|pqqI, pqqC, pqqL|yddC, pqqB|pqqG, pqqE, pqqD

#### 3.4.3 Stress response and resilience

Several genes related to mitigation of oxidative stress were identified in endophytic as well as in rhizospheric community. Glutathione s-transferase (gst) was identified in both communities. It plays a critical role in cellular detoxification by conjugating with harmful foreign and endogenous compounds. The root microbiome contained ssuD, oxyF and *bpe*T, while the rhizospheric microbiome contained antioxidant proteins thioredoxin (trxA, trxB) and quinone oxidoreductase (*Wrb*A). The organic peroxide sensor *ohr*R and regulator oxyR were identified in both rhizosphere and root microbiomes. Genes responsible for production of heat shock proteins (*hSP*s), hptG, and quinone oxidoreductase (*qor*) were identified in root microbiome. The genes encoding efflux pumps *zntA*, *cop*A, *czc*D responsible for eliminating toxic metals such as zinc, lead cadmium and copper were identified in root microbiome, along with the ars cluster. The rhizospheric microbiome possessed *znuABC*, a high affinity zinc binding transport system, alongside specific arsenic detoxification genes including the repressor *arsR*, the arsenate reductase *arsC*, and the arsenite-specific efflux pump *arsB*, as well as *cop*A/*ctp*A and *arsA*. Several types of genes that confer drug resistance to microbial species were identified in rhizosphere including *acr*A/*lir*/*mtc*A/mexA/*ade*I/*sme*D/*mtr*C/*cme*A. The metagenomic analysis of stress-related functional profile showed that the root microbiome features genes for heat shock response and distinct oxidative stress pathways, and the rhizospheric microbiome possesses an array of multidrug resistance genes alongside specific heavy metal detoxification systems (Table 4).

**TABLE 4:** Stress Response & Resilience genes predicted by PLaBAse functional annotation pipeline.

Root microbiome	Rhizosphere microbiome
Oxidative stress response genes oxyR, gst, bpeT, ssuD, oxyF, ohrR	Oxidative stress response genes: gst, trxA, wrbA, oxyR, trxB, ohrR
Heavy metal resistance genes: zntA, czcD/zitB/yrdO, laccase genes, ars, copA, zntA/C	Heavy metal resistance genes: copA/ctpA, arsA, znuA, znuB, znuC, arsB/arsC, arsR
Heat shock response genes: hsp, hptG, qor	Multidrug resistance gens: acrA/lir/mtcA/mexA/adeI/smeD/mtrC/cmeA, bpeT

#### 3.4.4 Other PGPTs

Several genes of central metabolic pathways were identified in both microbiomes (Table 5). High copy numbers and variety of sulphur metabolism genes were identified in rhizosperic microbial community, with many components were also present in root microbiome. Auxin synthesis and transport genes (*mia*A*, ipdC*, IAA-like and *log*) were identified predominantly in the endophytic root microbiome, with minor detection in soil microbiome. The genes associated with chemotaxis and motility were identified in both rhizosphere as well as in the endophytic microbial communities. Genes encoding enzymes for degradation of complex carbohydrates such as laccases, xylanases *(xlyAB)*, beta-glucosidases and chitinases were identified in endophytic and rhizosphere microbial communities. The root and rhizosphere microbiome also featured extensive iron acquisition systems and a strong genetic basis for stress mitigation through numerous efflux pumps, heavy metal resistance, and oxidative stress response genes. The prevalence of ABC transporters, PQQ biosynthesis genes, and motility/chemotaxis genes further highlighted the metabolic flexibility and dynamic colonization potential of the microbiome. Thus, a PLaBAse analysis of the *R. serpentina* root and rhizospheric soil microbiomes demonstrated that both communities are endowed with extensive plant growth-promoting traits, with distinct genetic architectures and functional emphases reflective of their ecological niches.

**TABLE 5:** Other PGPTs genes predicted by PLaBAse functional annotation pipeline.

Root microbiome	Rhizosphere microbiome
Sulfur metabolism: ssuD, cysJ, ssuA, ssuB, cysH, cysS, ssuE, cysK, cysK2	Sulfur metabolism: ssuD, cysJ, ssuA, ssuB, dmsB, cysH, cysM, cysS, ssuE, cysK, cysK2
ABC transporters: TC_AAT/yifK, ABC_SN_S, ABC_SN_A/ytlC, ddpA/ABC_PE_S, ddpD, ddpF, ABC_CD_P, ABC_CD_A, livK	ABC transporters: ABC_SN_S, ABC_SN_A/ytlC, ddpA/ABC_PE_S, ddpD, ABC_CD_P, ABC_CD_A, livK
Pyrroquiloline Quinone (PQQ) biosynthesis: pqqC, pqqD, pqqL/yddD	Pyrroquiloline Quinone (PQQ) biosynthesis: pqqC, pqqD, pqqL/yddC
Motility and chemotaxis: che, fli, pil families	Motility and chemotaxis genes: che, fli, pil gene families
Auxin pathways: miaA, ipdC, IAA like, log/yvdD, auxin symporter	Auxin pathways: miaA, log/yvdD
Carbon fixation: cbbR/cmpR/ndhR	Carbon fixation: cbbR/cmpR/ndhR
Fatty acid biosynthesis: ymfI/fabG/efpI	Fatty acid biosynthesis: ymfI/fabG/efpI
Dicarboxylate transport: dctA, dctM, actP	Dicarboxylate transport: dctA, dctM, actP
Central carbon metabolism: aceF, pgk, ppdK	Central carbon metabolism: aceF, pgk
Potassium uptake: trkD, trkD/kup	Potassium uptake: trkD/kup
Glycoside hydrolase: EC_3_2_1_67, EC_3_2_1_21, eng1	Not detected
Chitin degradation: chiA	Not detected
ATP synthase: atpA	ATP synthase: atpA
Complex carbohydrate degradation: xlyAB, beta-glucosidases, chitinases	Complex carbohydrate degradation: xlyAB, chitinases
Quorum sensing: ahlD	Quorum sensing: ahlD

**FIGURE 10:**
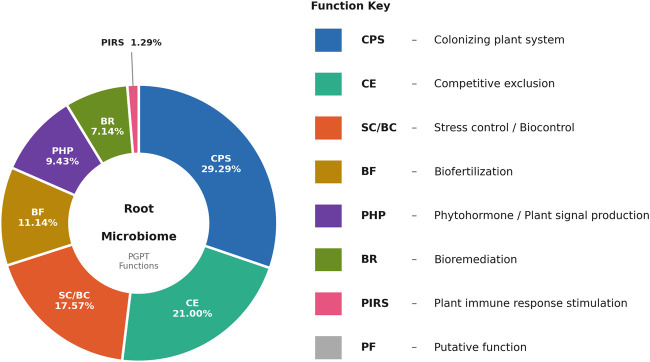
Plant growth promoting traits prediction by PLaBAse analysis of root microbiome.

**FIGURE 11:**
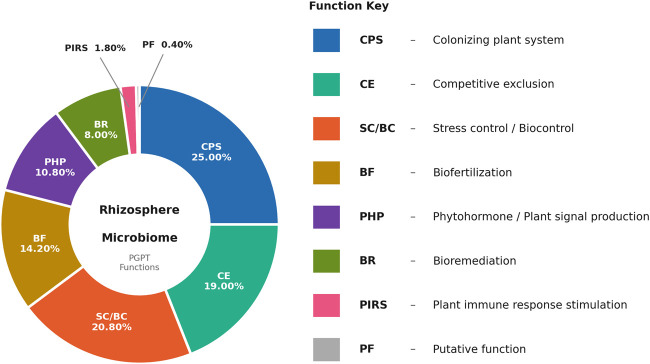
Plant growth promoting traits prediction by PLaBAse analysis of rhizosphere microbiome.

Collectively, while both microbiomes promote plant growth through stress resilience, nutrient acquisition, and hormone modulation, the root-associated community is more specialized for plant interaction and metabolic adaptability (Figure 10), and the rhizospheric soil community displays specific nutrient cycling capacities and stress tolerance mechanisms, alongside enhanced potential for dynamic colonization and environmental responsiveness (Figure 11). This functional partitioning underscores the roles of root and soil microbiomes in supporting *R. serpentina* health and productivity through synergistic plant-microbe and microbe-microbe interactions.

### 3.5 Secondary metabolite potential of microbial communities

Biosynthetic gene cluster profile identified through PRISM and antiSMASH (Skinnider et al., 2020; Blin et al., 2025) revealed a distinct functional divergence in the secondary metabolite potential of the *R. serpentina* root and rhizospheric soil microbiomes. This specialization appears to closely reflect the plant’s own capacity for producing pharmacologically significant alkaloids. The root-associated microbiome exhibits a broad repertoire of biosynthetic gene clusters (BGCs) that likely contribute to or modulate the plant’s chemical profile. These include diverse polyketide synthases (PKS), nonribosomal peptide synthases (NRPS), and hybrid NRPS-PKS clusters. Specifically, the presence of cyclopeptides from the NYH and XYP families, along with saccharide and terpene pathways, suggests a community uniquely adapted to the intimate chemical environment of the root and direct plant-microbe interactions (Table 6). The rhizospheric soil microbiome is enriched in BGCs associated with microbial competition, nutrient acquisition, and chemical signaling in the rhizosphere. This community is characterized by polyketide synthase clusters, NRPS-independent siderophore biosynthetic pathways, and ribosomally synthesized and post-translationally modified peptides (RiPPs)- such as lasso peptides and ranthipeptides (Table 6). The prominence of siderophores and lasso peptides highlights their critical roles in iron acquisition and competitive fitness, supporting plant health through indirect mechanisms. Collectively, these findings highlight that the root microbiome is specialized for intimate plant-microbe interactions, and the rhizospheric community is geared toward environmental adaptation. This functional differentiation in BGCs underscores the intricate interplay between *R. serpentina* and its associated microbiomes, suggesting that both communities contribute to the plant’s medicinal value and ecological fitness.

**TABLE 6:** Biosynthetic gene cluster (BGCs) predicted by antiSMASH analysis.

Predicted BGCs in root microbiome	Predicted BGCs in rhizospheric soil microbiome
Polyketide synthase: Trans-AT type I	Polyketide synthase: Type I
Polyketide synthase	Terpene
Non-ribosomal peptide synthase: Type I + polyketide synthase: Type I	Non-ribosomal peptide synthetase-independent siderophore
Non-ribosomal peptide synthase: Type I + polyketide synthase + saccharide	Recognition element-containing
Ribosomally synthesized and post-translationally modified peptides like	Ranthipeptide
Terpene: Monoterpene	Lassopeptide
Saccharide: II polysaccharide	-
Non-ribosomal peptide synthase: Type I + polyketide synthase: Type I + saccharide: Hybrid/tailoring	-

### 3.6 Resistome profile

ResFinder analysis of the *R. serpentina* root metagenome identified a limited but notable repertoire of antimicrobial resistance genes, predominantly conferring resistance to several classes of antibiotics. The resistome included *blaPAM-1* gene, which was detected in *Pseudomonas* and is associated with resistance to a broad spectrum of beta-lactam antibiotics including amoxicillin, ampicillin, multiple cephalosporins (cefepime, cefixime, Cefotaxime, cefoxitin, ceftazidime), carbapenems (ertapenem, imipenem, meropenem), and Piperacillin (with and without clavulanic acid/tazobactam). *blaZ* and *blaOXA-243* were also identified, conferring resistance to penicillins (amoxicillin, ampicillin, piperacillin, penicillin) and unknown beta-lactams, respectively. These beta lactam genes are typically associated with *pseudomonas* and related proteobacteria, which are well known for intrinsic and acquired beta-lactam resistance. Fosfomycin resistance was conferred by *fosB1* gene in *Bacillus*, while macrolide resistance was mediated by *mph*(K), also in *Bacillus,* predicting resistance to spiramycin and telithromycin. Chloramphenicol resistance was attributed to *catB1* in *Agrobacterium*. Tetracycline resistance was particularly extensive, with multiple genes in *Bacillus*: tet(45) and *tet(L), TOprJ4, tmexD4 and tmexD2*, collectively predicting resistance to tetracycline and doxycycline, and for *TOprJ4, tmexD4, and tmexD2*, also to minocycline and tigecycline. *Bacillus* is well documented reservoir of diverse tetracycline resistance mechanism in soil and plant associated microbiomes. Notably, no resistance genes were detected for aminoglycosides, glycopeptides, lincosamides, streptogramins, or several other antibiotic classes, suggesting a relatively narrow resistance profile within this root-associated microbial community. In contrast, ResFinder analysis of the *R. serpentina* rhizospheric soil metagenome revealed an absence of detectable acquired, clinically relevant antimicrobial resistance genes, likely because this natural, rhizosphere niche is less explored and harbour very low abundance of clinically annotated ARGS, whereas land-use conversion and antibiotic use in agriculture systems are known to expand soil resistome (Dungan et al., 2019; Ordine et al., 2023; Kiplimo et al., 2025). The absence of detectable ARGs in the soil microbiome bins, in contrast to their presence in root-associated bins, may reflect a true ecological difference in ARG reservoirs between the rhizosphere and bulk soil, or alternatively may result from technical and sampling limitations including low soil bin recovery, limited sequencing depth, and the restricted taxonomic representation of genome-resolved bins. Further studies with deeper sequencing and increased biological replication are needed to determine whether this absence reflects a genuine biological pattern or an artifact of incomplete community recovery (Von Wintersdorff et al., 2016; Gao et al., 2023).

## 4 Discussion

The present metagenomic study of the root and rhizosphere microbiomes of *R. serpentina* reinforces and extends our current understanding of plant-associated microbial communities. Consistent with previous reports on medicinal plants and model species, our data revealed a strong bacterial dominance in both compartments, with Proteobacteria, Firmicutes, and Actinobacteria as the most abundant phyla, a pattern also observed in the rhizospheres of *Arabidopsis thaliana*, maize, and other medicinal plants (Bulgarelli et al., 2012; Reid et al., 2021). Notably, Actinobacteria, particularly the order Streptomycetales and genus *Streptomyces*, were highly prevalent in both root and soil samples, aligning with their established roles as keystone taxa for rhizosphere health and plant growth promotion. This is further supported by recent studies isolating endophytic and rhizospheric bacteria from *R. serpentina* and demonstrating their capacity for secondary metabolite production and plant growth promotion (Lata and Gond, 2025). This enrichment may be driven by root exudates, which act as carbon sources and signalling molecules that shape the rhizosphere microbiota by selectively recruiting beneficial taxa (Bais et al., 2006; Badri and Vivanco, 2009). The enrichment of *Pseudomonas, Bacillus and Agrobacterium* in *R. serpentina*, together with genes for chemotaxis, motility, and hormone modulation, may reflect exudate driven selection of taxa capable of colonizing root and providing plant growth promoting functions (Zhang et al., 2014; Comeau et al., 2021; Yang et al., 2025). This pattern is consistent with current models of microbiome assembly, where bulk soil act as a species pool, the rhizosphere is enriched by root-derived substrates, and the endosphere represent a highly selective habitat along filtering continuum (Edwards et al., 2015; Li et al., 2025).

Functionally, both root- and soil-derived metagenomes in our study harboured extensive plant growth-promoting traits, including genes for nitrogen fixation, phosphate solubilization, siderophore-mediated iron acquisition, and stress tolerance. These findings parallel the functional versatility described in other rhizosphere studies of medicinal plants (Trivedi et al., 2020; Saleem et al., 2021; Jabborova et al., 2024). The phosphorous is an essential nutrient for plant development; however, in Indian soils, only about 1% of the total soil phosphorous is available for direct plant uptake (Sharma et al., 2013). The metagenomic signatures obtained from the *R.* serpentina microbiome effectively elucidate the metabolic synergy among these phosphate solubilizing and mineralizing microorganisms. While the microbial communities in rhizosphere are exposed to the complex, competitive and dynamic soil niche, the endophytic community establishes a specialized relationship inside the host plant. Thus, metagenomic data illustrates redundancy of some functions that must be significant to continue during the times of nutritional limitation and stress. However, our data highlights distinct functional profile within the rhizosphere microbiome, characterized by notable metabolic diversity and stress resilience, as evidenced by wide spectrum of nitrogen transformation pathways and heavy metal resistance genes. This is consistent with the enhanced functional diversity and adaptability reported for rhizosphere communities (Edwards et al., 2015; Bahram et al., 2018). Ecologically, this suggests rhizosphere community function as a buffer zone that mobilizes nutrients and mitigates environmental stresses, while the root microbiome is more helpful in hormone balance, nutrient assimilation, and oxidative stress management (Bai et al., 2022). Thus, the root microbiome was enriched in genes for plant hormone modulation and intimate plant-microbe interactions, supporting the “filtering continuum” model of microbiome assembly (Fitzpatrick et al., 2018; Bulgarelli et al., 2013; Edwards et al., 2015).

Archaeal taxa were detected predominantly in the rhizosphere and were nearly absent in root tissues, corroborating observations from other plant systems that suggest selective exclusion of archaea from the endosphere (Taffner et al., 2020). Additionally, our analysis of biosynthetic gene clusters revealed that root-associated bins were enriched in nonribosomal peptide synthetase and polyketide synthase clusters, and the rhizosphere was characterized by a higher abundance of siderophore and lasso peptide gene clusters. Several studies on bio-informatic analysis and gene knockout revealed that biosynthetic gene clusters (BGCs) like polyketide synthases (PKS), nonribosomal peptide synthases (NRPS), and hybrid PKS-NRPS clusters contribute to the production of alkaloids, as demonstrated in studies on naphthoquinone-oxindole alkaloids, indole alkaloids, and other hybrid compounds from various microbial and fungal sources (Mollah et al., 2020; Lin et al., 2021).

Collectively, these results reinforce and extend current models of rhizosphere-root microbiome structure and function, while highlighting the unique taxonomic and functional signatures of the *R. serpentina* microbiome. It suggests root exudation and host filtering mechanisms play important role in selecting microbial taxa with complementary functions in nutrient cycling, stress tolerance, and secondary metabolite production, thereby supporting both plant health and biosynthesis of pharmaceutically relevant compounds. This has important implications for the development of targeted bioinoculants and the discovery of novel bioactive compounds, as also suggested by recent reviews (Shenya et al., 2025). Experimental validation of plant-microbe interactions and the bioactivity of novel compounds identified from BGCs will be crucial for translating these findings into practical applications, such as the development of targeted bioinoculants or new pharmaceuticals derived from the *R. serpentina* microbiome. Moreover, future studies should incorporate deeper sequencing and different sequencing technologies such as long read sequencing with more replicates will enhance recovery and would also provide statistical insights.

## 5 Conclusion

This comprehensive metagenomic investigation of the root and rhizosphere microbiomes of *R. serpentina* reveals a highly diverse and functionally robust microbial community. The subtle differences in community composition highlight the ecological specialization and functional complementarity of these plant-associated microhabitats. Functional gene profiling demonstrated that both communities are equipped with extensive plant growth-promoting traits, positioning them as promising reservoirs for bioinoculant development. The discovery of diverse and novel biosynthetic gene clusters further underscores the biotechnological potential of these microbiomes for the discovery of new bioactive compounds, offering an alternative to traditional plant-based compounds. This study provides valuable insights into the taxonomic and functional landscape of the *R. serpentina* microbiome. These findings lay the groundwork for future research integrating multi-omics approaches and experimental validation to harness the ecological and pharmaceutical potential of plant-associated microbial communities in sustainable agriculture and drug discovery.

## Data Availability

The datasets presented in this study can be found in online repositories. The names of the repository/repositories and accession number(s) can be found in the article/Supplementary Material.
