# Supplementary material for: From diversity to function: microbiome-mediated plant growth promotion, secondary metabolism, and antimicrobial resistance in Rauwolfia serpentina
Source: Front Bioinform. 2026 Jul 14;6:1796770. doi: 10.3389/fbinf.2026.1796770 (PMC13408031; doi:10.3389/fbinf.2026.1796770)
Supplement: Supplementary file 1 [file DataSheet1.zip › Final_Supplementary material Frontiers in Bioinformatics/Supplementary Table 1.docx]

**Root microbiome**

| **Bins** | **Total length** | **Num contigs** | **N50** | **GC content** | **Percent completion** | **Percent redundancy** |
| --- | --- | --- | --- | --- | --- | --- |
| Bin_1 | 6511067 | 2701 | 2709 | 40.16475613 | 100 | 1.408450704 |
| Bin_2 | 12079333 | 1793 | 20862 | 64.4297719 | 80.28169014 | 19.71830986 |
| Bin_3 | 24249381 | 6750 | 5188 | 58.69322477 | 100 | 487.3239437 |
| Bin_4 | 64948277 | 23314 | 3466 | 38.92839278 | 60.56338028 | 74.64788732 |
| Bin_5 | 8262053 | 5114 | 1581 | 44.07978484 | 47.88732394 | 9.85915493 |
| Bin_6 | 59800963 | 25275 | 2906 | 35.31271683 | 15.6626506 | 3.614457831 |
| Bin_7 | 44155366 | 21520 | 2202 | 34.16052992 | 43.66197183 | 14.08450704 |

**Rhizosphere microbiome**

| **Bins** | **Total length** | **Num contigs** | **N50** | **GC content** | **Percent completion** | **Percent redundancy** |
| --- | --- | --- | --- | --- | --- | --- |
| Bin_1 | 2085032 | 956 | 2435 | 46.5901731 | 100 | 23.94366197 |
| Bin_2 | 2930108 | 1727 | 1619 | 56.7848618 | 82.89473684 | 14.47368421 |
| Bin_3 | 5644518 | 4092 | 1321 | 65.76983357 | 69.01408451 | 53.52112676 |
| Bin_4 | 1080967 | 691 | 1588 | 51.05858428 | 67.10526316 | 17.10526316 |
| Bin_5 | 1148245 | 778 | 1429 | 42.96153241 | 0 | 0 |
