# Supplementary figures and images for: From diversity to function: microbiome-mediated plant growth promotion, secondary metabolism, and antimicrobial resistance in Rauwolfia serpentina
Source: Front Bioinform. 2026 Jul 14;6:1796770. doi: 10.3389/fbinf.2026.1796770 (PMC13408031; doi:10.3389/fbinf.2026.1796770)

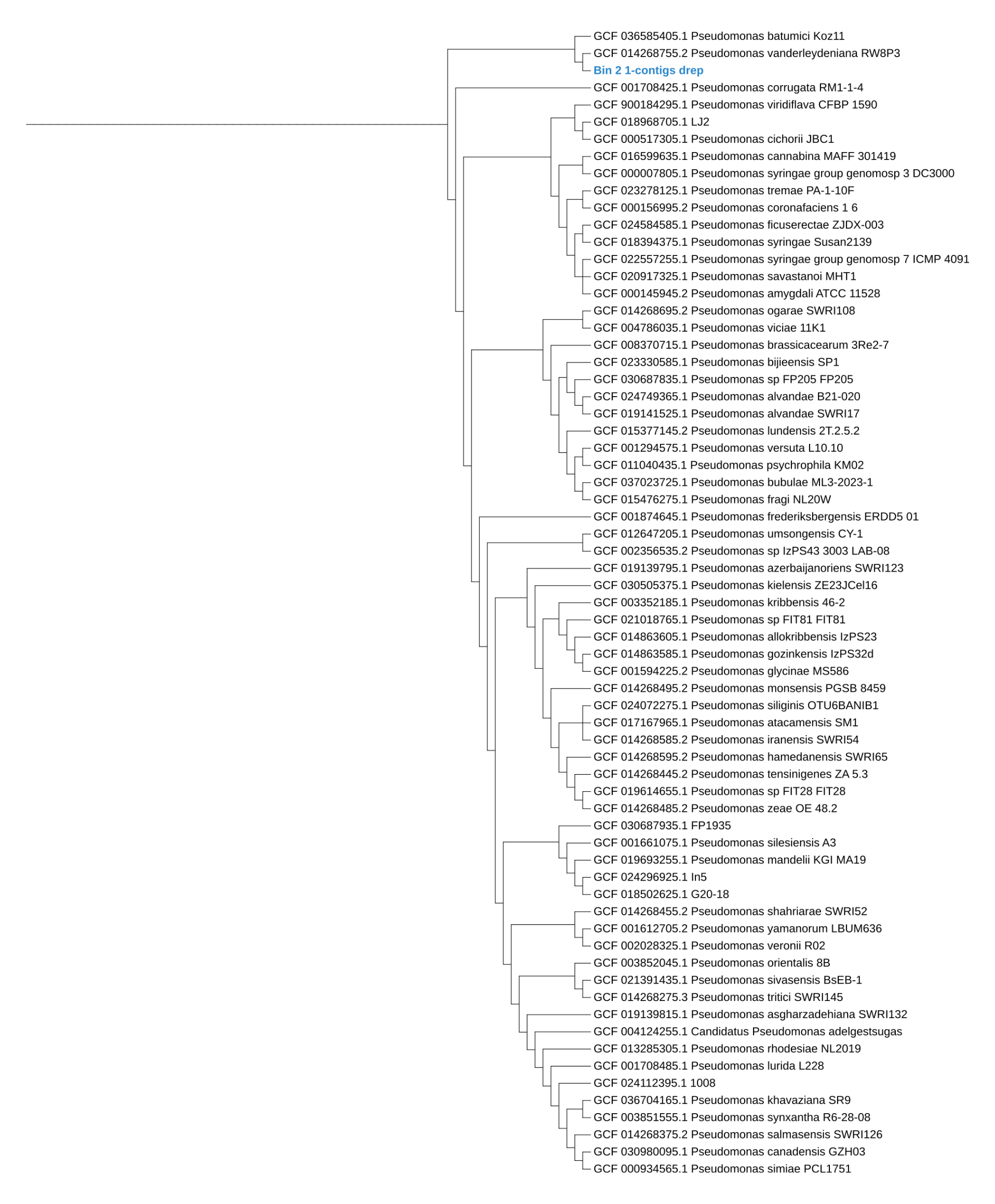

Supplement: Supplementary file 1 [file DataSheet1.zip › Final_Supplementary material Frontiers in Bioinformatics/Root microbiome- Phylogenomics images/Root Microbiome Bin2.png]

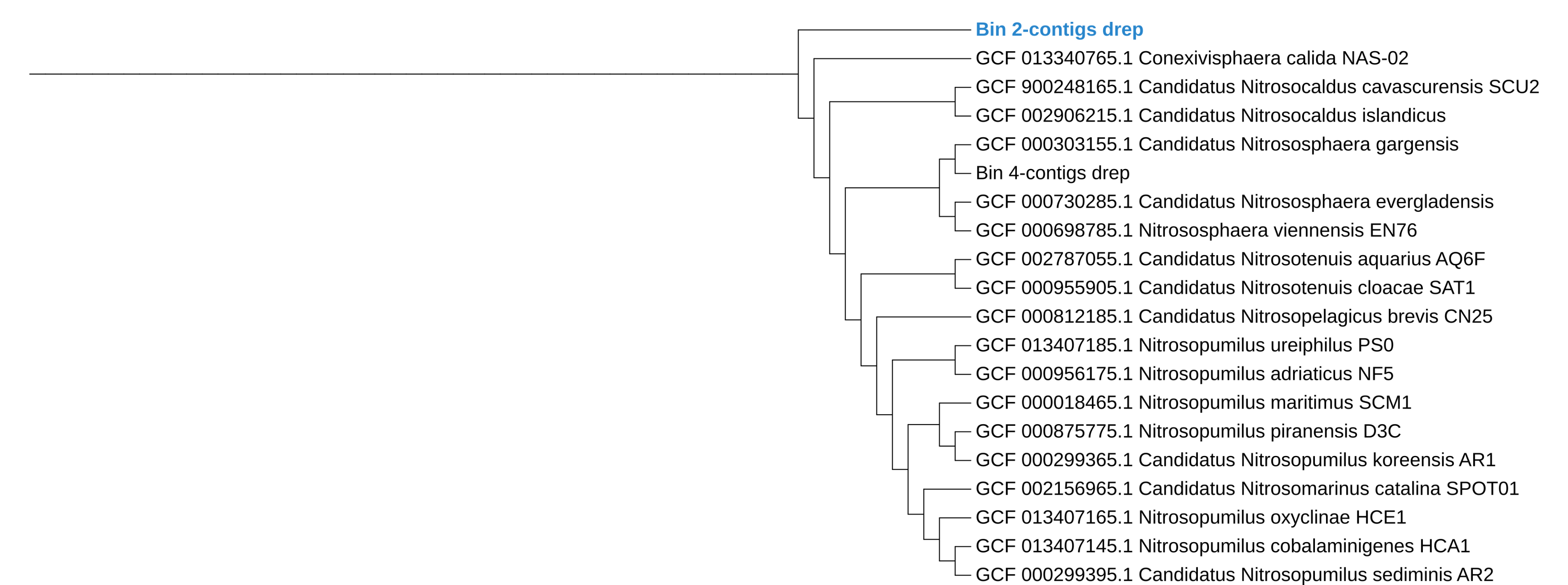

Supplement: Supplementary file 1 [file DataSheet1.zip › Final_Supplementary material Frontiers in Bioinformatics/Rhizospheric soil microbiome-Phylogenomics images/Soil Microbiome Bin2.png]

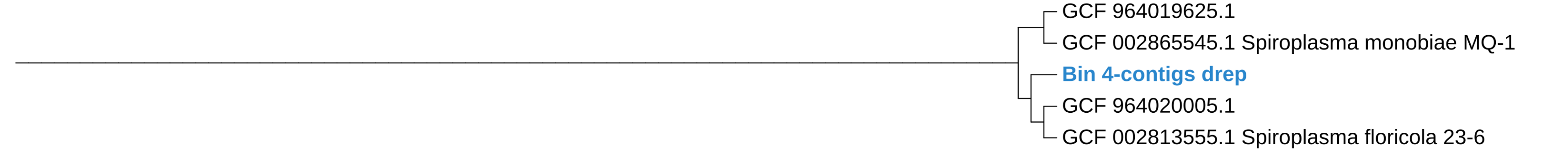

Supplement: Supplementary file 1 [file DataSheet1.zip › Final_Supplementary material Frontiers in Bioinformatics/Root microbiome- Phylogenomics images/Root Microbiome Bin3.png]

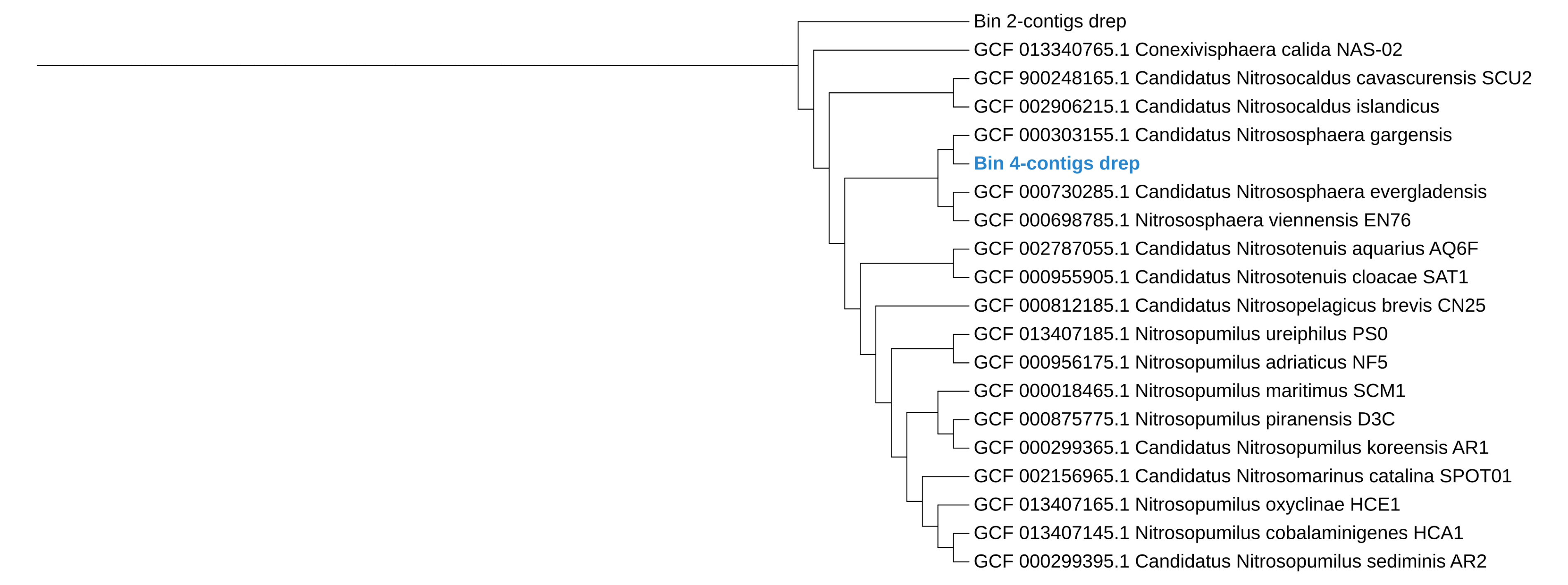

Supplement: Supplementary file 1 [file DataSheet1.zip › Final_Supplementary material Frontiers in Bioinformatics/Rhizospheric soil microbiome-Phylogenomics images/Soil Microbiome Bin4.png]

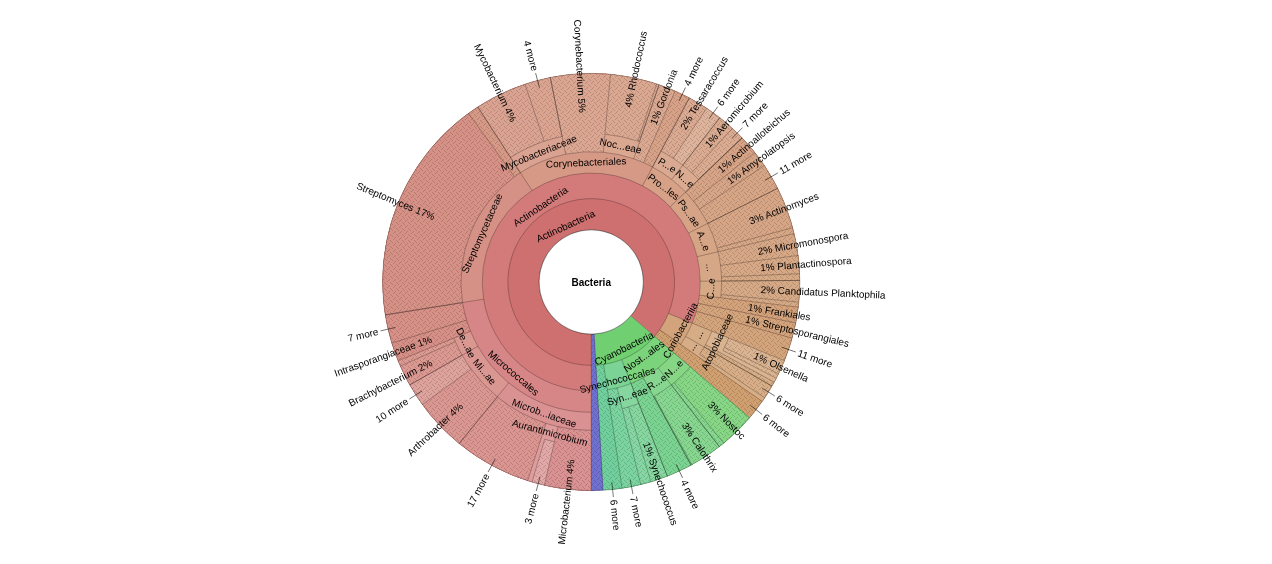

Supplement: Supplementary file 1 [file DataSheet1.zip › Final_Supplementary material Frontiers in Bioinformatics/Supplementary figure 1A- Krona plot- Root microbiome.png]

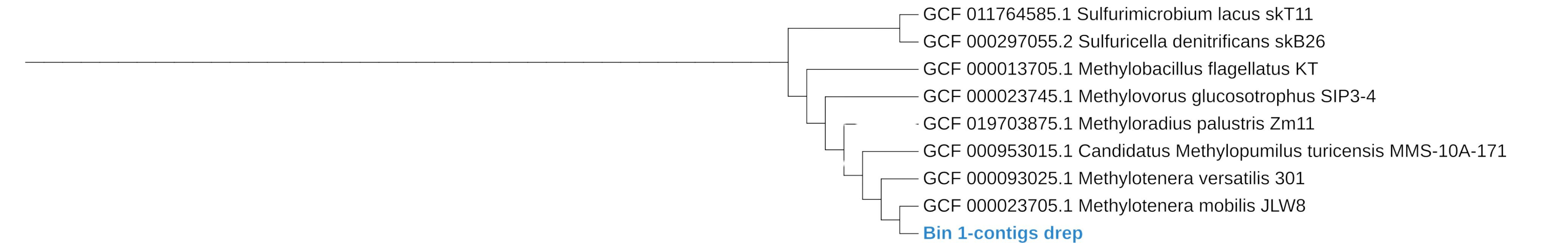

Supplement: Supplementary file 1 [file DataSheet1.zip › Final_Supplementary material Frontiers in Bioinformatics/Rhizospheric soil microbiome-Phylogenomics images/Soil Microbiome Bin1.png]

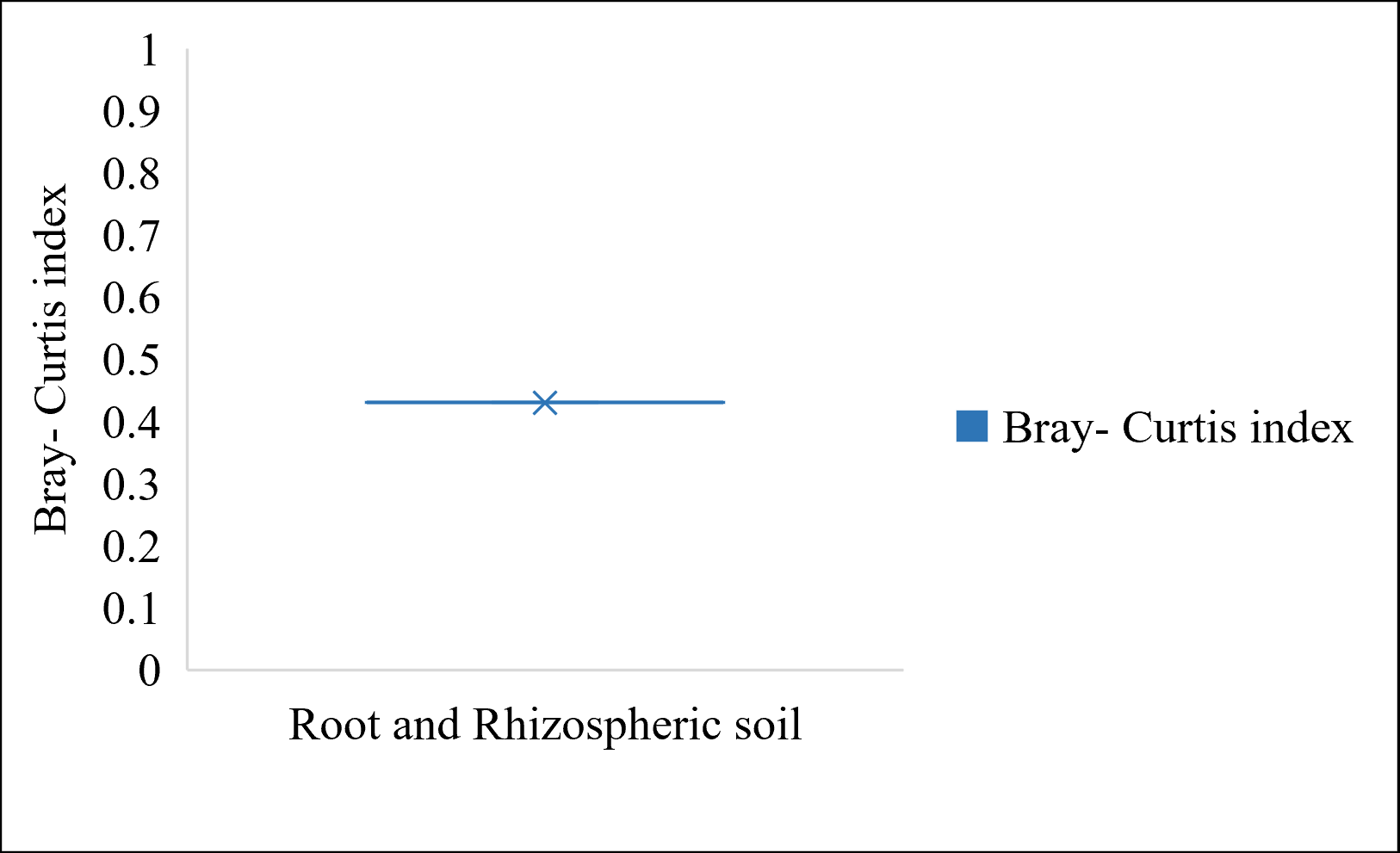

Supplement: Supplementary file 1 [file DataSheet1.zip › Final_Supplementary material Frontiers in Bioinformatics/Supplementary figure 2B .png]

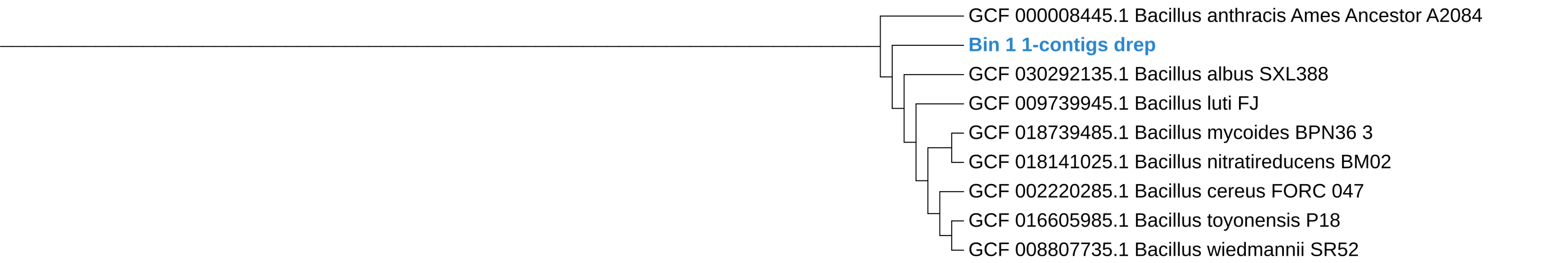

Supplement: Supplementary file 1 [file DataSheet1.zip › Final_Supplementary material Frontiers in Bioinformatics/Root microbiome- Phylogenomics images/Root Microbiome Bin1.png]

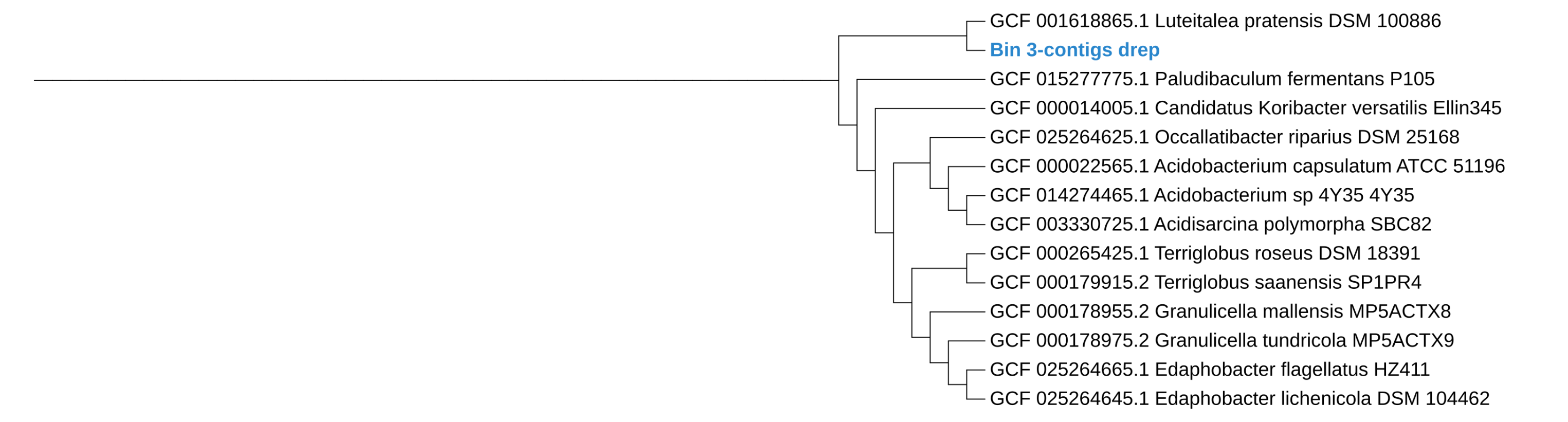

Supplement: Supplementary file 1 [file DataSheet1.zip › Final_Supplementary material Frontiers in Bioinformatics/Rhizospheric soil microbiome-Phylogenomics images/Soil Microbiome Bin3.png]

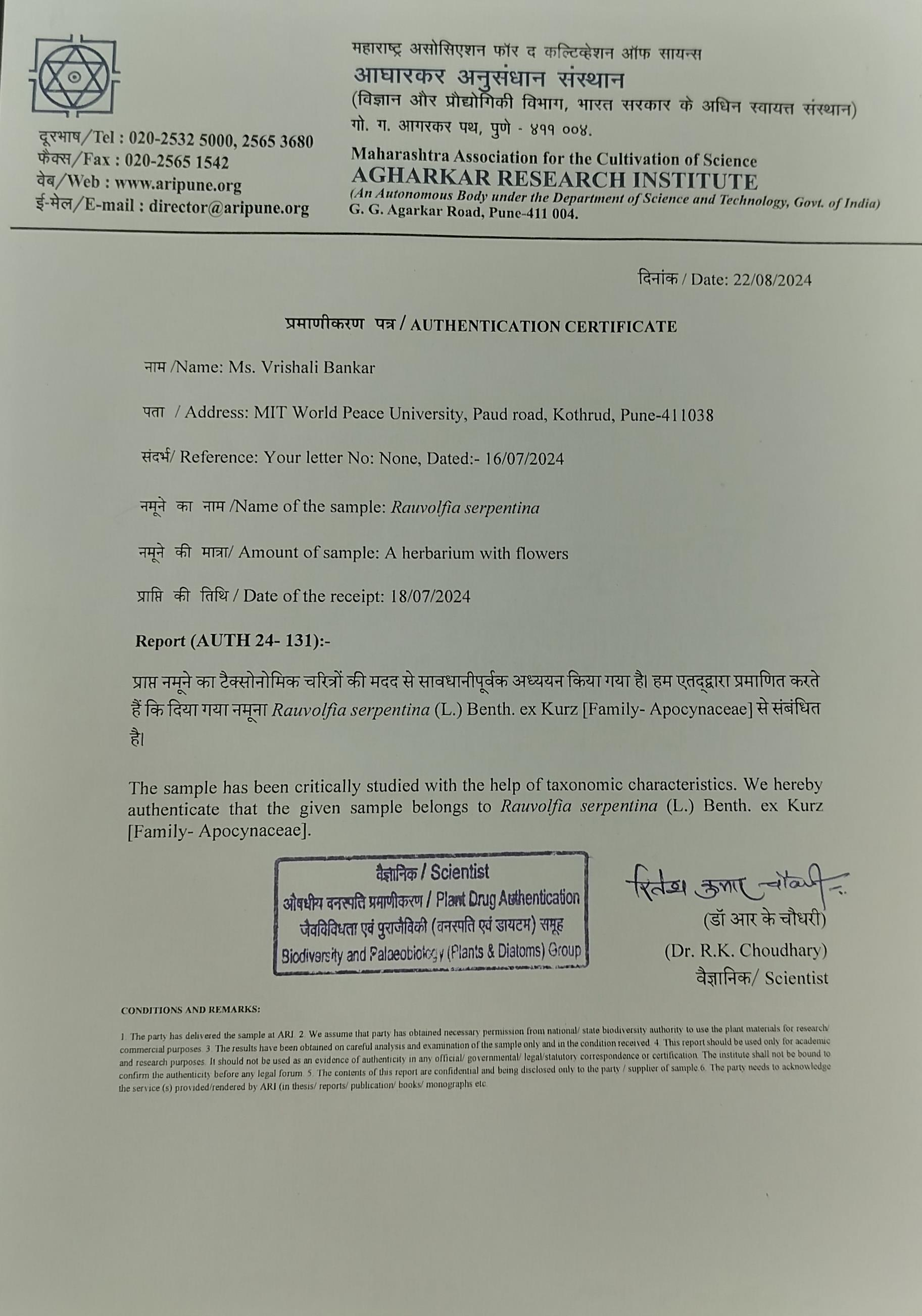

Supplement: Supplementary file 1 [file DataSheet1.zip › Final_Supplementary material Frontiers in Bioinformatics/Authentication certificate.png]

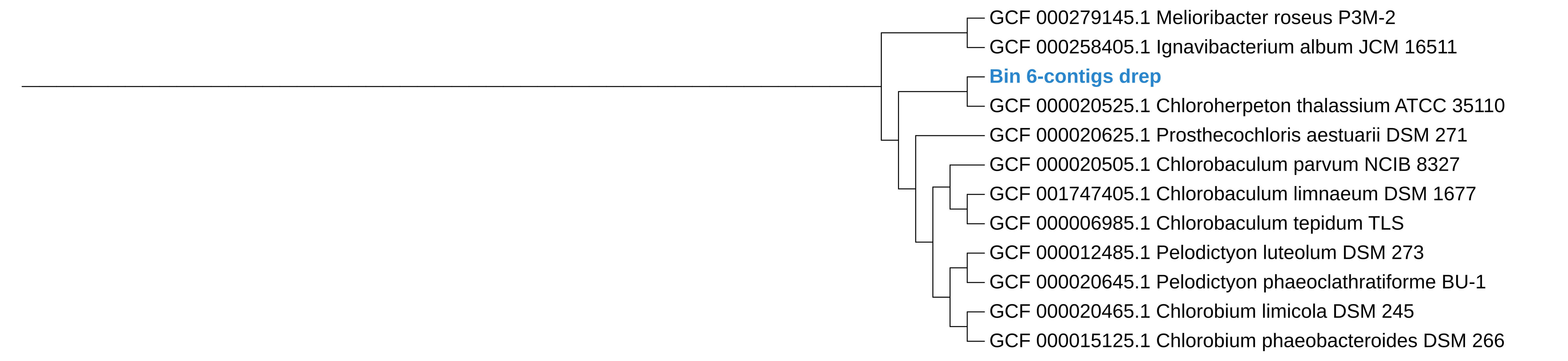

Supplement: Supplementary file 1 [file DataSheet1.zip › Final_Supplementary material Frontiers in Bioinformatics/Root microbiome- Phylogenomics images/Root Microbiome Bin5.png]

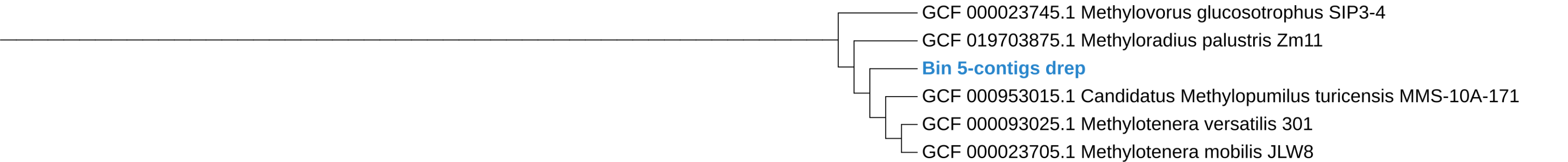

Supplement: Supplementary file 1 [file DataSheet1.zip › Final_Supplementary material Frontiers in Bioinformatics/Root microbiome- Phylogenomics images/Root Microbiome Bin4.png]

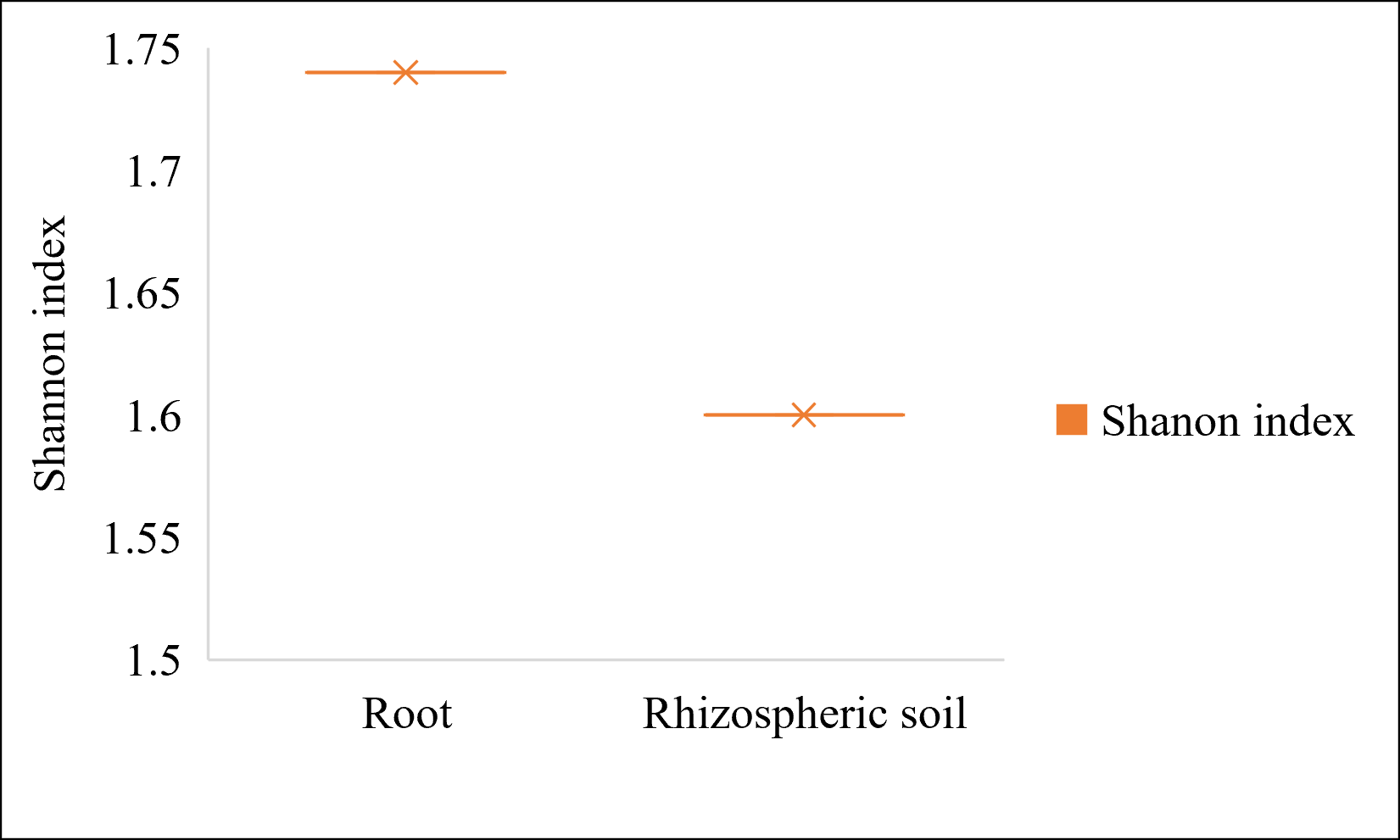

Supplement: Supplementary file 1 [file DataSheet1.zip › Final_Supplementary material Frontiers in Bioinformatics/Supplementary figure 2A.png]

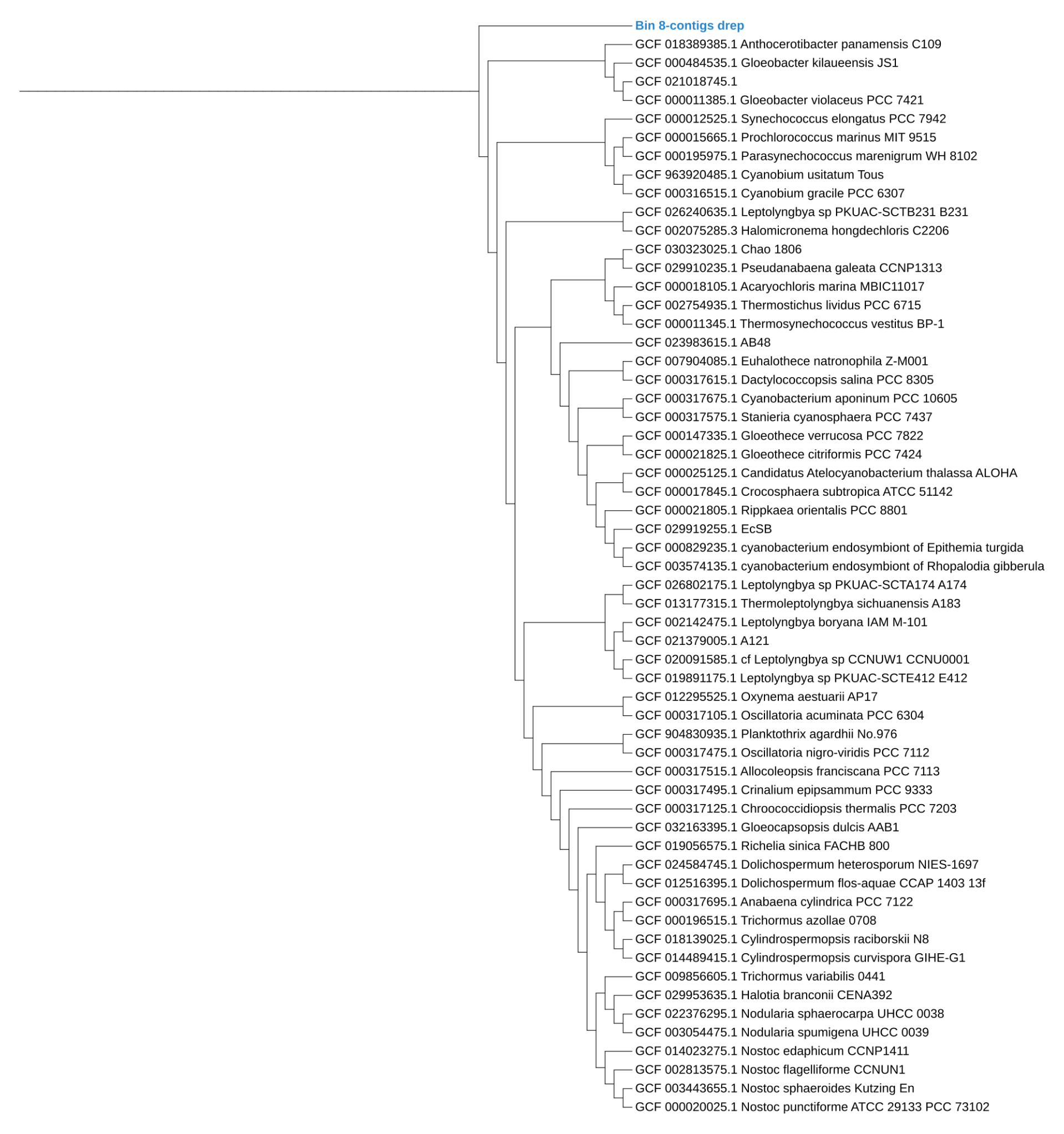

Supplement: Supplementary file 1 [file DataSheet1.zip › Final_Supplementary material Frontiers in Bioinformatics/Root microbiome- Phylogenomics images/Root Microbiome Bin6.png]
